# Key role for Kv11.1 (ether‐a‐go‐go related gene) channels in rat bladder contractility

**DOI:** 10.14814/phy2.15583

**Published:** 2023-02-07

**Authors:** Vincenzo Barrese, Zena Wehbe, Alice Linden, Sarah McDowell, Elizabeth Forrester, Oleksander Povstyan, Karen D. McCloskey, Iain A. Greenwood

**Affiliations:** ^1^ Vascular Biology Research Centre Molecular and Clinical Sciences Research Institute, St George's University of London London UK; ^2^ Department of Neuroscience, Reproductive Sciences and Dentistry University of Naples Federico II Naples Italy; ^3^ Patrick G. Johnston Centre for Cancer Research, School of Medicine, Dentistry and Biomedical Sciences Queen's University Belfast Belfast UK; ^4^ Institute of Medicine University of Leeds Leeds UK

**Keywords:** BK channels, detrusor smooth muscle, ether‐a‐go‐go‐related genes, *kcnh2* gene, Kv11.1

## Abstract

In addition, to their established role in cardiac myocytes and neurons, ion channels encoded by ether‐a‐go‐go‐related genes (ERG1‐3 or *kcnh2,3* and *6*) (*kcnh*2) are functionally relevant in phasic smooth muscle. The aim of the study was to determine the expression and functional impact of ERG expression products in rat urinary bladder smooth muscle using quantitative polymerase chain reaction, immunocytochemistry, whole‐cell patch‐clamp and isometric tension recording. *kcnh*2 was expressed in rat bladder, whereas *kcnh*6 and *kcnh*3 expression were negligible. Immunofluorescence for the *kcnh2* expression product Kv11.1 was detected in the membrane of isolated smooth muscle cells. Potassium currents with voltage‐dependent characteristics consistent with Kv11.1 channels and sensitive to the specific blocker E4031 (1 μM) were recorded from isolated detrusor smooth muscles. Disabling Kv11.1 activity with specific blockers (E4031 and dofetilide, 0.2–20 μM) augmented spontaneous contractions to a greater extent than BK_Ca_ channel blockers, enhanced carbachol‐driven activity, increased nerve stimulation‐mediated contractions, and impaired β‐adrenoceptor‐mediated inhibitory responses. These data establish for the first time that Kv11.1 channels are key determinants of contractility in rat detrusor smooth muscle.

## Graphical abstract

Our data establish for the first time that Kv11.1 channels are key determinants of contractility in rat detrusor smooth muscle. Disabling Kv11.1 activity with specific blockers augmented spontaneous contractions to a greater extent than BKCa channel blockers, enhanced carbachol‐driven activity, increased nerve stimulation‐mediated contractions, and impaired beta‐adrenoceptor‐mediated inhibitory responses.

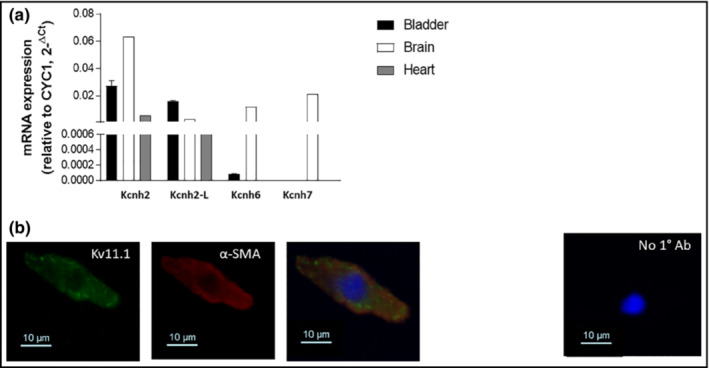

## 1 INTRODUCTION

The ether‐a‐go‐go‐related gene (ERG) family comprises three members in humans, *ERG1,2,3* (*KCNH2*, *KCNH6*, and *KCNH7*, HUGO gene nomenclature) that encode for tetrameric voltage‐dependent potassium channels (Kv11.1–Kv11.3, respectively) (Gutman et al., 2005). Kv11 channels exhibit distinctive voltage‐dependent kinetics due to a dominant C‐type inactivation that is relieved quicker than channel deactivation by membrane hyperpolarization (Dai & Zagotta, 2017; Hoshi & Armstrong, 2013; Spector et al., 1996).

*KCNH2* is expressed predominantly in cardiac myocytes and congenital mutations in this gene result in gross perturbation of the normal cardiac electrical activity associated with hereditary long QT syndrome (London et al., 1997; Vandenberg et al., 2001). *KCNH6* and *KCNH7* expression are restricted mainly to neuronal cells, where the expression products contribute to resting membrane conductance (Bauer & Schwarz, 2018). However, several studies have identified Kv11 channels as important dampeners of cellular excitability in several smooth muscles. *KCNH2* expression has been detected by qPCR and immunocytochemistry in rodent stomach, portal vein, and myometrium (Greenwood et al., 2009; Ohya, Asakura, et al., 2002; Ohya, Horowitz, et al., 2002). Moreover, currents with kinetics characteristic of Kv11 channels have been recorded in opossum esophagus, mouse portal vein, and nonpregnant mouse and human myometrium (Akbarali et al., 1999; Greenwood et al., 2009; Ohya, Horowitz, et al., 2002; Parkington et al., 2014; Yeung & Greenwood, 2007). In addition, selective Kv11 channel blockers like E4031 or dofetilide depolarise membrane potential and increase contractility in opossum esophagus, rat stomach, mouse gall bladder, equine and human jejunum, mouse portal vein, bovine epididymis, mouse and human myometrium (Akbarali et al., 1999; Farrelly et al., 2003; Greenwood et al., 2009; Lillich et al., 2003; Mewe et al., 2008; Ohya, Asakura, et al., 2002; Ohya, Horowitz, et al., 2002; Parkington et al., 2014; Parr et al., 2003; Yeung & Greenwood, 2007). Common to all these smooth muscles is the exhibition of spontaneous, phasic contractile behavior associated with action potential discharge.

The detrusor smooth muscle of the urinary bladder also exhibits spontaneous contractions which, in tension recordings from bladder strips, manifest as low‐amplitude contractions in contrast to much larger nerve‐evoked or agonist‐evoked (e.g., acetylcholine or ATP) contractions (Drake et al., 2018). The genesis of spontaneous contractions is a source of debate with both inherent myogenicity and urothelial‐derived or mucosal mediators being implicated (Brading, 1997; Drake et al., 2018). Their role is considered to provide a degree of tone and dynamic adjustment of optimal bladder shape during filling so that the bladder can be effectively emptied from any volume (Turner & Brading, 1997). During bladder filling, mechanisms that limit the amplitude of spontaneous contractions are essential for the bladder to expand and act as a reservoir. Detrusor smooth muscle expresses a panel of potassium channels that act as brakes on contractility through repolarization of the action potential and control of the resting membrane potential (Petkov, 2011). Of the myriad potassium channels, there is substantial evidence for the functional expression of BK (Petkov, 2014), Kv (Maylz & Petkov, 2020), and Kv7 channels (Anderson et al., 2013; Bientinesi et al., 2017; Provence et al., 2018; Svalo et al., 2015) and their contribution to spontaneous contractions. There is a paucity of work supporting the expression of *ERG* in a bladder and to the best of our knowledge, only one paper on ERG channel function where increased spontaneous contraction amplitude and corresponding decreased frequency in guinea‐pig bladder strips by the ERG channel blocker, E4031 was reported (Imai et al., 2001). The goal of the present study was to determine the molecular expression of *Kcnh* genes in rat detrusor smooth muscle and to ascertain the functional impact of ERG channels in detrusor contractility using various pharmacological selective modulators.

## 2 MATERIALS AND METHODS

### 2.1 Ex vivo bladder preparation techniques

Whole bladders were dissected from male Wistar rats (200–250 g) killed by cervical dislocation in accordance with the 1986 UK Animals Scientific Procedures Act (1986). Bladders were dissected free of fat and connective tissue then cut into transverse bands of 1 mm thickness from the mid‐region of the bladder, while submerged in an ice‐cold physiological saline solution (PSS) of the following composition (mmol/L); 1.25 CaCl_2_, 5 glucose, 25 NaHCO_3_, 1.18 NaH_2_PO_4_, 1.17 MgSO_4_, 4.5 KCl, 119 NaCl. The bands were then mounted on micro‐pins within the tissue chambers of a myograph capable of isometric tension recording. Bands were set to an initial tension of 2 mN and reset to this level over a 1‐h equilibration period. Tissue viability was then assessed by a 60 mM KCl challenge. The effect of two structurally different, open channel blockers of Kv11.1, E4031 and dofetilide (Spector et al., 1996) was investigated using single concentrations per tissue. In a separate experimental series, carbachol was added cumulatively to all bladder bands. After the washout of carbachol, tissue bands were incubated with one of the following agents: dimethyl sulphoxide (DMSO) (0.2%) as the vehicle control or 2 μM E4031 and 20 μM E4031.

For isoprenaline experiments, the level of spontaneous activity was enhanced by the application of 20 mM KCl. Isoprenaline was applied cumulatively from 0.001 to 1 μM. After washout, tissue bands were preincubated with one of the following channel modulators: 20 μM E4031, 10 μM XE991 (Kv7 channel blocker),100 nM Iberiotoxin (BK_Ca_ blocker), or DMSO as vehicle control (0.2%) and isoprenaline was re‐applied.

All drugs were obtained from HelloBio except iberiotoxin (Alomone).

For electrical field stimulation (EFS) studies, transverse bands (1 mm thickness) were mounted in vertical baths attached to force transducers via wire hooks. EFS was delivered via silver/silver chloride electrodes placed at the top and bottom of the vertical organ baths using a Grass stimulator (Grass) and the following parameters, 0.3 ms pulse width, 10 s duration, 30–40 V, 0.5–32 Hz frequency range. Organ baths were perfused with oxygenated PSS (solution as above) at 37°C at a rate of 2–3 mL/min; drugs were delivered via the perfusion system. Recordings were made via an AD/DA converter (National Instruments) and a personal computer running Chart software (University of Strathclyde, Dr J Dempster).

Contractions were measured in Clampfit (pClamp software; Axon Instruments) and data were collated in Excel (Microsoft Office) and analyzed in GraphPad Prism software. EFS contraction amplitudes were normalized to a control contraction evoked by 60 mM K^+^ Krebs solution for each experiment. Data are presented as mean ± standard error of the mean (SEM), and data sets were tested with two‐way analysis of variance (ANOVA) and Bonferroni post‐hoc tests with *p* < 0.05 considered significant.

Spontaneous contractions were recorded using LabChart software. The parameters that were then measured and analyzed, included baseline tone, amplitude and frequency of individual phasic contractions, and maximum change in contractility. Baseline tone is the lowest point of the trace from which phasic contractions develop. Maximum change in baseline contractility was measured for the highest crest amplitude and compared with the initial baseline tone. Amplitude was calculated as the average force (mN) of the peaks of the amplitudes 2 min prior to the addition of the next drug. Frequency was calculated as the average number of individual contractions occurring per minute.

### 2.2 Quantitative real‐time polymerase chain reaction assay

cDNA was produced by reverse transcription of messenger RNA extracted from the intact bladder tissue samples. Quantitative real‐time polymerase chain reaction (qPCR) was performed using specific primers for rat *Kcnh*2, *Kcnh*2‐L, *Kcnh*6, and *Kcnh*7 (Table 1) and SYBR Green Master Mix from Primer design. Two isoforms are known for Kcnh2 (erg1) gene: a full‐length isoform, encoding a protein composed of 1163 amino acids (previously termed erg1 long or erg1a), and a short one (erg1 short or erg1b), missing the first 5 exons of full‐length Erg1 and encoding an 821 amino acid protein with a different N‐terminus with respect to Erg1a, encoded by an alternative exon 5 (Vandenberg et al., 2012).

**TABLE 1:** Primers used for qPCR experiments.

Gene	*Kcnh2*	*Kcnh2‐L*	*Kcnh6*	*Kcnh7*	*cyc1*
Primer sequence Forward	5′‐CCCCTCCATCAAGGACAAGT‐3′	5′‐CCTCGACACCATCATCCGCA‐3′	5′‐GTGGATGTGGTCCCTGTGAA‐3′	5′‐GCCCGGGCTCAACCTGAAGA‐3′	5′‐TCGAAAACGCATGGGACTCA‐3′
Primer sequence Reverse	5′‐TGAGCATGACACAGATGGAG‐3′	5′‐AGGAAATCGCAGGTGCAGGG‐3′	5′‐AGAGCCCAGGAAGCTGTGTG‐3′	5′‐TGGCCTGGATGTCCGTTGTC‐3′	5′‐TGACCACTTATGCCGCTTCA‐3′
Amplicon size (base pairs)	128	156	141	169	85
Target sequence region	2145–2272	369–533	328–468	3012–3180	889–973

Therefore, to evaluate the expression in rat bladder of the different isoforms, we used two sets of primers targeting the *Kcnh2* gene; these sequences were designed to amplify a common region of Kcnh2 found in the two transcripts variant (here defined as “*Kcnh2* primers”), and a specific sequence that can be found only in the longer variant of the same gene (here called “*Kcnh2‐L* primers”).

The reaction was carried out in a clean, designated environment to minimize the risk of contamination. Primer and SYBR Green Mix were prepared and added to a 96‐well plate, and the prepared cDNA template was added. Control wells containing no cDNA were used to detect the possible occurrence of contamination of the samples. qPCR reaction was performed using a CFX96 Real‐Time PCR machine (Biorad) according to the following protocol: 95°C for 10 min (enzyme activation), then 40 cycles at 95°C for 15 s and 60°C for 60 s (denaturation and data collection). A melt curve to check for nonspecific PCR products was performed at the end of the reaction. Results were then collected and analyzed using BioRad CFX Manager Software. Data were normalized to the housekeeping gene Cytochrome C1 (CYC1) and expressed using the 2^−ΔCt^ formula.

### 2.3 Electrophysiology

Single smooth muscle cells (SMCs) were isolated from the whole bladder by enzymatic dispersal. Tissues were carefully cleaned of the mucosal layer (comprising urothelium, lamina propria, small blood vessels, and connective tissues) and bathed for 10 min in nominally Ca^2+^‐free PSS. The vessels were then incubated at 37°C in Ca^2+^‐free PSS‐containing collagenase type IA (2 mg/mL) and protease type X (1 mg/mL) for 25–30 min followed by a 10 min wash in Ca^2+^‐free PSS at room temperature (RT). Single cells were liberated by gentle mechanical agitation of tissues with a wide‐bore Pasteur pipette and the suspension was transferred to experimental chambers. All experiments were performed at RT using the whole‐cell patch‐clamp technique. The solution used for recordings was of the following composition (mM): KCl 60, NaCl 66, MgCl_2_ 1.2, CaCl_2_ 0.1, d‐glucose 12, and HEPES 10, pH was adjusted to 7.35 with NaOH. The solution was supplemented with paxilline (1 μM) and nicardipine (5 μM). Patch pipettes were fire‐polished and had resistances of 4–8 MΩ when filled with the pipette solution containing (in mmol/L): 110 K gluconate, 30 KCl, 0.5 MgCl_2_, 5 HEPES, and 0.5 EGTA. The electrical signals were recorded using an Axopatch 200B patch‐clamp amplifier (Molecular Devices). Electrical signals were generated and digitized at 1 kHz using a Digidata 1322A hosted by a PC running pClamp 9.0 software (Axon Instruments). Data were analyzed and plotted using pClamp and MicroCal Origin software. All data are presented as mean ± SEM. To isolate Kv11 channel currents, we employed a protocol used previously (Greenwood et al., 2009; Ohya, Asakura, et al., 2002) that took advantage of the biophysical properties of the channel. Cells were held at 0 mV, where Kv11.1 channels were in an inactive state and then stepped to hyperpolarized potentials from −120 to −20 mV every 5 s. Steps to hyperpolarized voltages remove the inactivation that is followed by channel closure (deactivation). This results in a distinctive “hooked” appearance as both the recovery from inactivation and deactivation is faster at more hyperpolarized potentials (Ohya, Horowitz, et al., 2002; Smith et al., 1996).

### 2.4 Immunocytochemistry

Bladder SMC were fixed with 3% paraformaldehyde solution at 22–24°C (RT) for 10 min, treated with 0.1 M glycine in phosphate‐buffered saline (PBS) for 5 min, and incubated in blocking solution (PBS containing 0.1% Triton X‐100 and 1% bovine serum albumin) for 1 h at RT. Subsequently, cells were incubated overnight at 4°C with goat polyclonal anti‐HERG antibody (dilution 1:100), which had been validated previously using overexpression systems (Barrese et al., 2017) now discontinued or with mouse monoclonal anti‐HERG antibody (1:100; sc‐377388), and mouse monoclonal anti‐α‐smooth muscle actin antibody (dilution 1:1000) (Cat.#: A5228; Sigma). Samples were washed with PBS and incubated for 1 h with donkey antigoat secondary antibody conjugated to Alexa Fluor 568 (Cat.#: A‐11057) and donkey antimouse secondary antibody conjugated to Alexa Fluor 488 (Cat.#: A32766) (dilution 1:100) (ThermoFisher). All antibodies were diluted in the blocking solution. No primary antibody was used as a negative control. Coverslips were mounted on glass slides; images were acquired using a Nikon A1R confocal microscope (Nikon Instruments Europe BV).

### 2.5 Data analysis

Data were presented as mean ± SEM. For most graphical presentations, data were normalized and given as a percentage increase from resting conditions or as a percentage difference from the control group. Data processing and presentation utilized Microsoft Excel and Prism GraphPad software. Statistical analysis was carried out with one‐way and two‐way ANOVAs, where statistically significant values *, **, ***, **** represent *p* < 0.05, *p* < 0.01, *p* < 0.001, *p* < 0.0001, respectively. The number of tissues utilized in an experiment is referred to as “*n*,” whereas the number of rats culled is denoted by “*N*.”

## 3 RESULTS

### 3.1 Kv11.1 transcript (Kcnh) is expressed in rat detrusor smooth muscle

**FIGURE 1:**
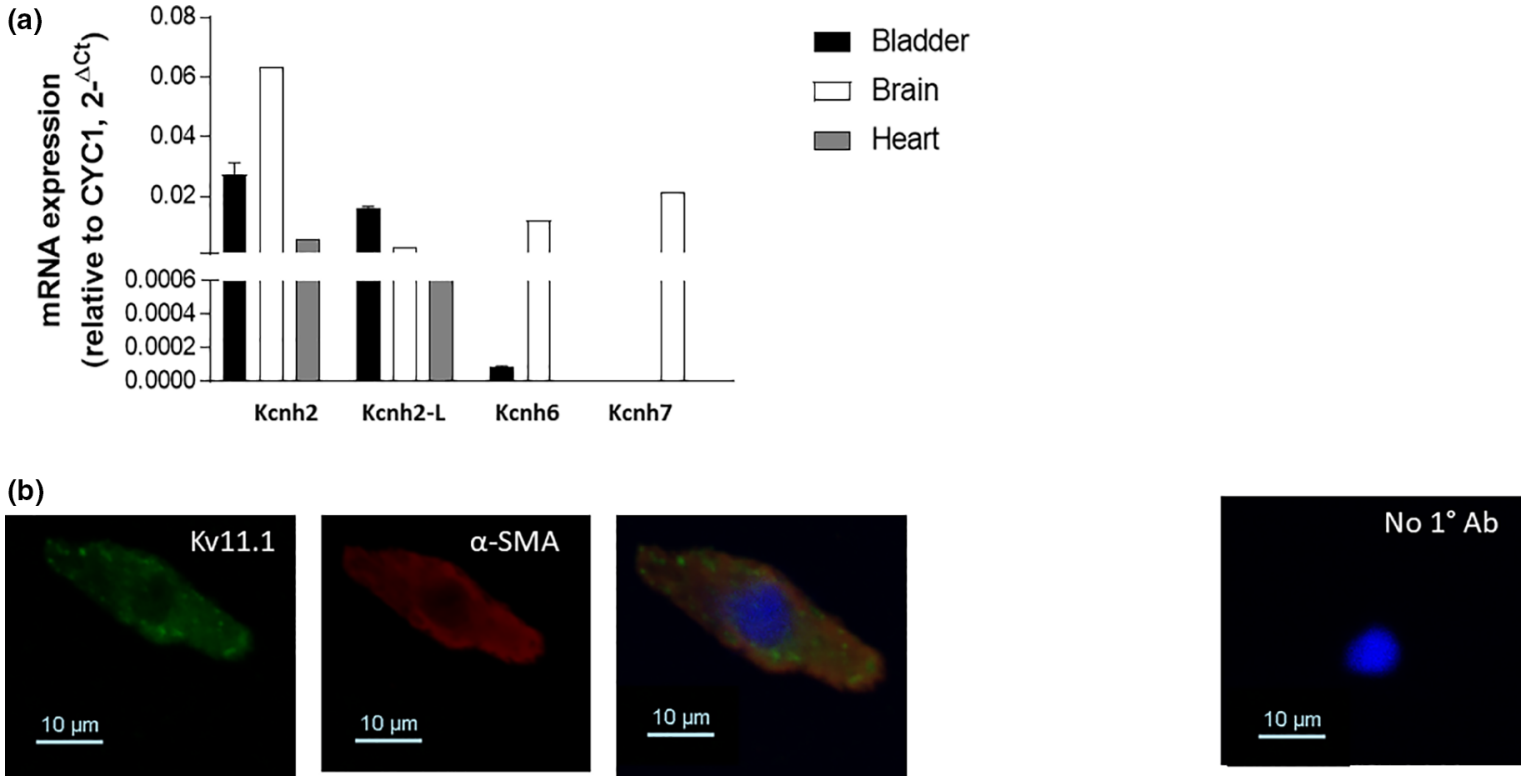
Expression of Kcnh2 channels in rat detrusor. (a) Quantitative PCR experiments showing the expression of total Kcnh2, long Kcnh2 isoform (Kcnq2‐L), Kcnh6 and Kcnh7 mRNAs in rat detrusor, normalized to the housekeeping gene Cyc1. The rat brain and heart were used as positive controls. Data are expressed as mean ± standard error of the mean. *N* = 5. (b) Fluorescent images of smooth muscle cells isolated from rat detrusor and labeled with a mouse monoclonal Kv11.1 antibody (left column) and α‐smooth muscle actin (α‐SMA, middle column). Cells where primary antibodies were omitted (incubated with secondary antibodies only) are also shown (right panel). Each image is representative of five separate dispersals. Scale bar = 10 μm.

qPCR using primers specific for all three *Kcnh* isoforms revealed robust expression of *Kcnh*2 but the minimal expression of Kcnh6 and Kcnh7 in the intact rat detrusor (Figure 1a). Of the two known variants of *Kcnh*2 (London et al., 1997), the rat detrusor predominantly expressed the longer form (Figure 1a). Immunofluorescence experiments using anti‐Kv11.1 antibodies were undertaken to assess the expression of *Kcnh*2 products in detrusor muscle. SMCs were identified by co‐staining with α‐smooth muscle actin. In these cells, anti‐Kv11.1 immunofluorescence showed peripheral distribution, indicative of plasma membrane localization of Kv11.1 channels (Figure 1b). Similar staining was seen with the goat anti‐HERG polyclonal validated by Barrese et al. (2017).

**FIGURE 2:**
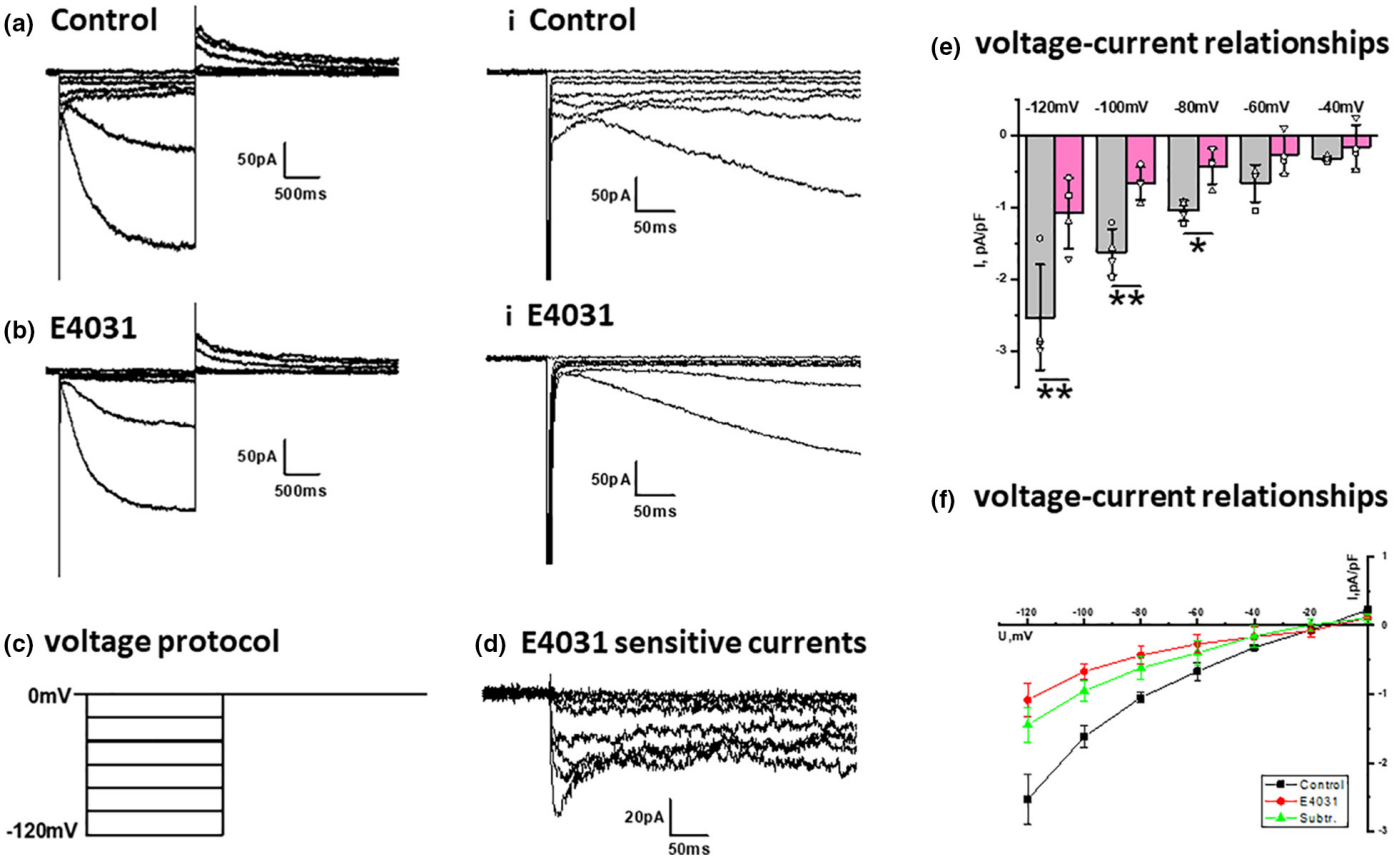
Whole‐cell electrophysiology of isolated detrusor smooth muscle cells. (a, b) Show currents evoked by the protocol shown in (c) in the absence (a) and presence (b) of E4031 (2 μM). The right‐hand panels (a.i, b.i) show a zoom of the initial 200 ms of the test step to highlight the E4031‐sensitive current. (d) Shows a representative set of E4031‐sensitive currents. (e, f) Show the mean current amplitude at different test potentials in the absence and presence of E4031. Data were taken from 8 to 12 cells from four animals (*N* = 4, *p < 0.05, **p < 0.01, one‐way analysis of variance, error bars represent standard error of the mean).

Whole‐cell electrophysiology was performed to determine if functional Kv11.1 currents could be recorded from isolated detrusor SMC using a protocol utilized previously (Greenwood et al., 2009; Ohya, Horowitz, et al., 2002) that takes advantage of the distinctive properties of the Kv11.1 channel. When isolated detrusor SMC were hyperpolarized from a holding potential to −120 mV a large, slowly developing inward current was recorded. This current was the HCN‐encoded mixed cation current known as the “funny” current that is responsible for sino‐atrial node pacing and that has been characterized in rat bladder and mouse portal vein previously (Green et al., 1996; Greenwood & Prestwich, 2002). The HCN channel current was preceded by an inward current with a prominent “hook” (Figure 2) that decayed rapidly and was abrogated by the Kv11.1‐specific blocker E4031 (Figure 2a–f) (*N* = 4, *p* < 0.05). The kinetics of the development and subsequent decay of the E4031‐sensitive current were voltage‐dependent becoming slower at less negative potentials (Figure 2d). These kinetics are consistent with the voltage‐dependent properties of inactivation recovery and deactivation exhibited by Kv11.1 channels (Ohya, Horowitz, et al., 2002; Spector et al., 1996).

### 3.2 Kv11.1 blockers affect detrusor contractility

**FIGURE 3:**
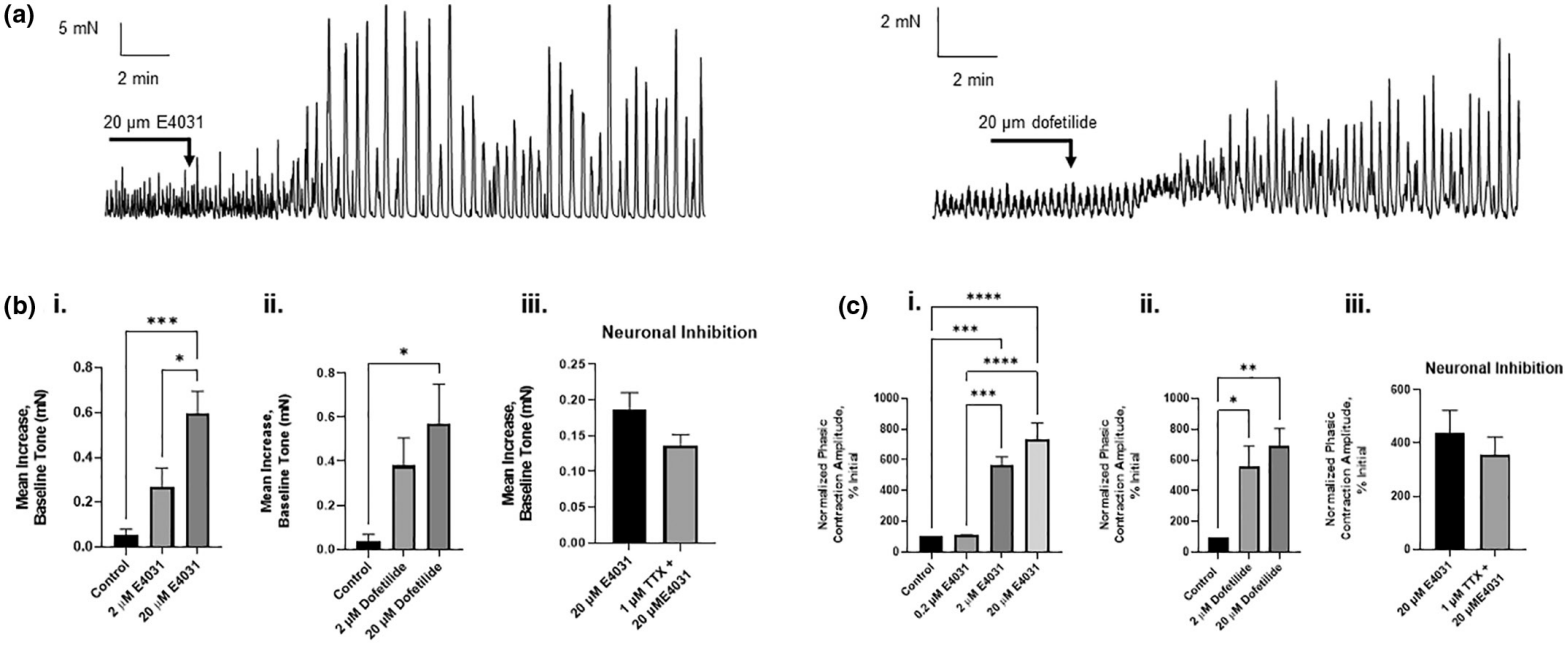
Effect of Kv11.1 channel inhibitors on spontaneous contractile activity. (a) Representative trace of basal tone and phasic contraction amplitudes after the addition of 20 μM of the Kv11.1 inhibitors, E4031 (left) and Dofetilide (right). (b) The maximum increase in baseline tone at various concentrations of Kv11.1 inhibitors E4031 (i) and dofetilide (ii), and in the presence of neuronal inhibition with TTX (iii) (*N* = 5). (c) Percent increase in the amplitude of phasic contractions compared with the control, in the presence of Kv11.1 inhibitors, E4031 (i) and dofetilide (ii), and in the presence of neuronal inhibition (iii) (*N* = 5) (*p < 0.05, **p < 0.01, ***p < 0.001, ****p < 0.0001, one‐way analysis of variance, error bars represent standard error of the mean).

Transverse bands of the bladder exhibited spontaneous phasic contractile activity that was sustained through the duration of typical recordings. The addition of two structurally different Kv11.1‐specific blockers, E4031 and dofetilide (Yeung & Greenwood, 2007), augmented detrusor contractility considerably in a concentration‐dependent manner (Figure 3). In an intact bladder (in which the mucosa is retained), application of 2 μM E4031 or 2 μM dofetilide did not increase basal tone, whereas 20 μM E4031 caused a significant increase (+0.6 ± 0.1, *N* = 5, *p* < 0.001), as did 20 μM dofetilide (+0.56 ± 0.18, *N* = 5, *p* < 0.05) (Figure 3b.i,ii). To confirm if neuronal input modulated the effect of E4031 on baseline tone, 1 μM of tetrodotoxin (TTX) was added prior to 20 μM E4031 to prevent neurotransmitter release. E4031 still increased baseline tone even in the presence of TTX (+0.14 ± 0.01, *N* = 5) and this was not significantly different from its corresponding control (Figure 3b.iii).

Phasic contraction amplitude was significantly increased in the presence of 2 μM E4031 (566.7% ± 54.21%, *N* = 5, *p* < 0.001) and 20 μM E4031 (734.5% ± 106.8%, *N* = 5, *p* < 0.0001) in an intact detrusor (Figure 3c.i). Similarly, 2 and 20 μM dofetilide also increased phasic contraction amplitude by 560.4% (±131.7%, *N* = 5, *p* < 0.05) and 695.1% (±110.1%, *N* = 5, *p* < 0.01), respectively (Figure 3c.ii). This heightened contractile activity was unaltered over a 5‐h experimental period. E4031 still increased the amplitude of spontaneous contractions in the presence of TTX (+355.2% ± 67.9, *N* = 5) (Figure 3c.iii). These data show that Kv11.1 channels have a considerable impact on spontaneous activity in the rat detrusor and that this effect is not likely mediated via neuronal stimulation.

### 3.3 Kv11.1 blockers and carbachol‐mediated contractions

**FIGURE 4:**
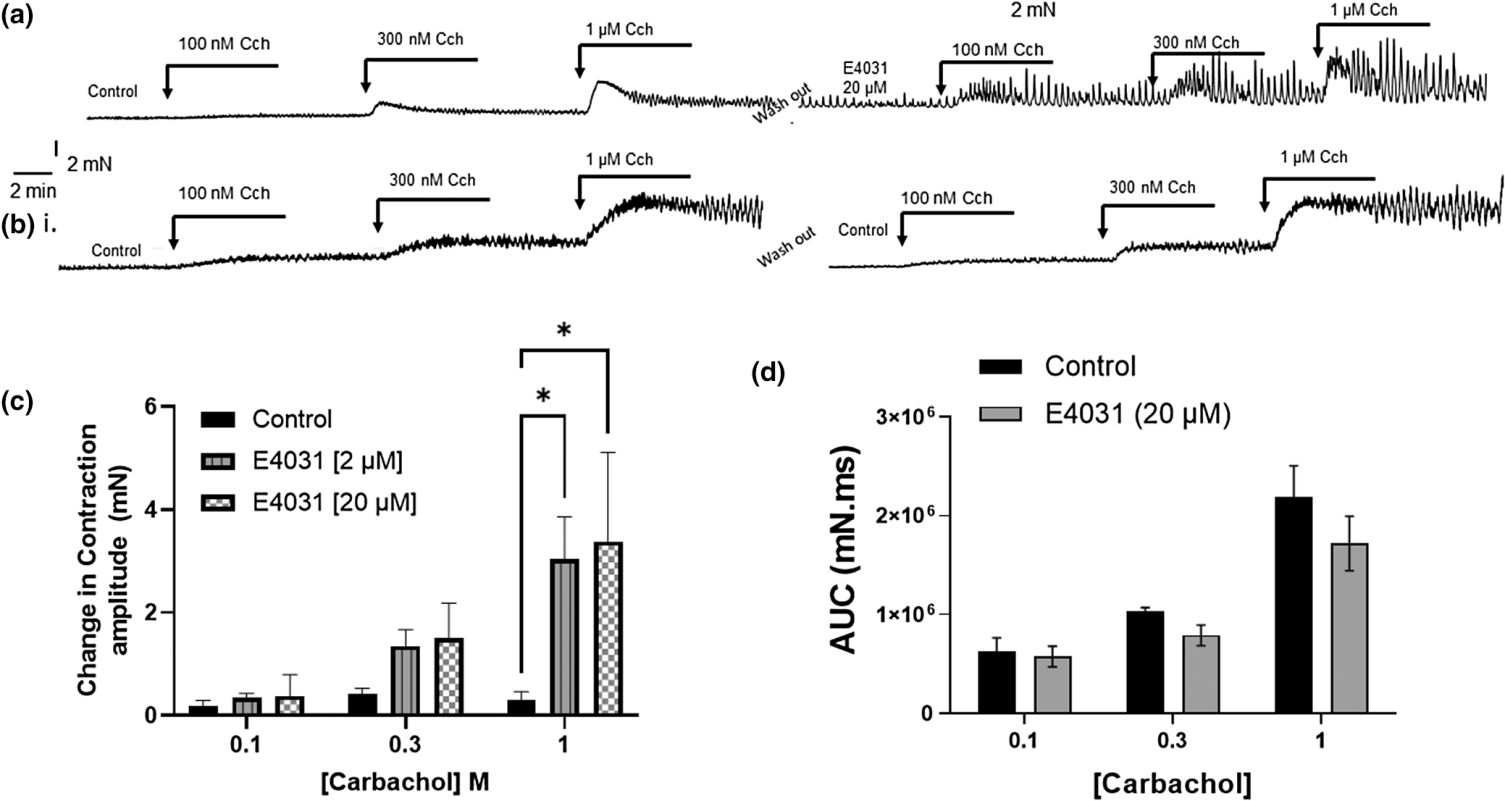
Assessing amplitude of phasic contractions in response to carbachol in the presence and absence of potassium channel inhibitors. (a) Representative traces of contraction amplitudes in response to carbachol before and after the addition of the Kv11.1 inhibitor, E4031 (20 μM) within the same tissue (internal control). (b) Representative traces of contraction amplitudes in response to carbachol before and after the addition of vehicle control (dimethyl sulphoxide). (c) The change in the mean amplitude of contractions taken over a 2‐min period when the effect of Cch reaches a plateau and prior to the addition of the next concentration. The greatest change in amplitude, compared with control was observed in the presence of E4031 with 1 μM CCh (*N* = 5), *p < 0.05, error bars represent SEM; statistical analysis using two‐way ANOVA, with Bonferroni multiple comparison test. (d) The area under the curve analysis carried out at the maximum change in baseline tone after the addition of each concentration of Cch, in the presence of 20 μM E4031 (*N* = 5; two‐way ANOVA; error bars represent SEM). ANOVA, analysis of variance; SEM, standard error of the mean.

Application of the muscarinic receptor agonist, carbachol (1–10 μM) (CCh) evoked concentration‐dependent contractions that were manifest as enhanced baseline superimposed by individual contractions (Figure 4a,b). All bladder strips were exposed to increasing concentrations of CCh followed by a washout with PSS and this served as the internal control for each strip. The washout was followed by a second application of CCh either with the prior incubation with 2 μM, 20 μM E4031, or vehicle control. The subsequent increase in baseline tone, contractile amplitude, or area under the curve was measured as the change from its internal control (first exposure to CCh). Preapplication of E4031 (2 μM) enhanced the effect of CCh on the amplitude of phasic contractions and further enhancement was observed with 20 μM (Figure 4c). Neither concentration of E4031 affected the force integral of the carbachol response measured as the area under curve during the first 3 min of the CCh response (Figure 4d).

### 3.4 Kv11.1 blockers and nerve‐mediated contractions

**FIGURE 5:**
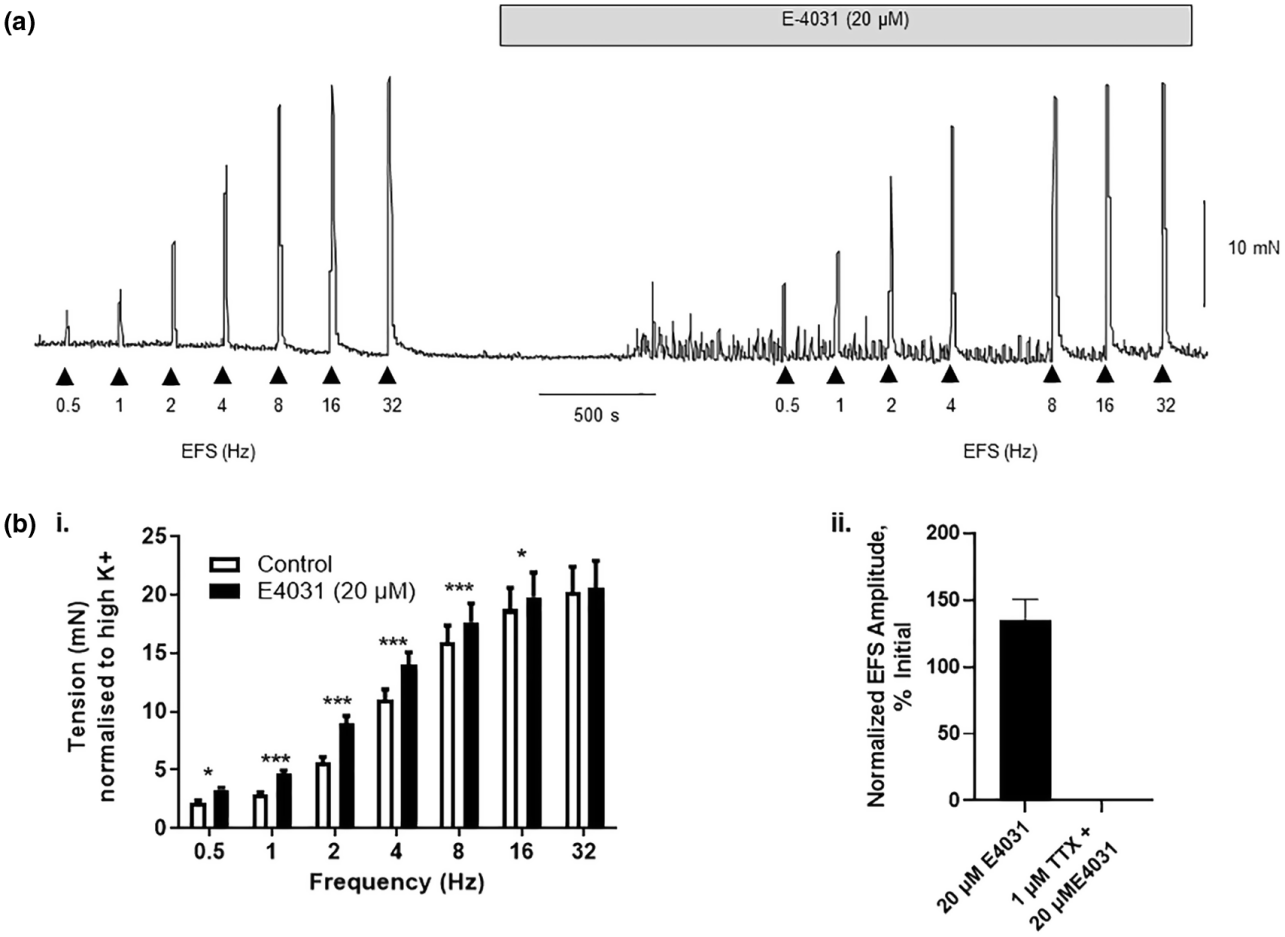
Effect of E‐4031 on nerve‐evoked contractions. (a) Electrical field stimulation (EFS) across the frequency range 0.5, 1, 2, 4, 8, 16, and 32 Hz evoked frequency‐dependent contractions. Following treatment with E4031 (20 μM), the stimulation protocol was repeated. Note the enhancement of spontaneous contractions in the presence of E4031. (b.i) Summary data from experimental series (N = 5), normalized to high K+ (60 mM) contraction. (b.ii) Mean data showing the effect of E4031 on EFS (30 Hz, 30 V) with and without prior incubation with TTX (*t* test). *p < 0.05, ***p < 0.0001; error bars represent standard error of the mean. (Two‐way analysis of variance with Bonferroni post‐hoc test).

Electrical field stimulation (EFS, 0.5–32 Hz) evoked frequency‐dependent contractions (Figure 5a), which were inhibited by the voltage‐gated sodium channel blocker tetrodotoxin (see Campbell et al., 2017). E4031 (20 μM) caused small but significant increases in EFS contraction amplitude across all frequencies (0.5, 1, 2, 4, 8, and 16 Hz; *p* < 0.05, *N* = 5) (Figure 5b.i). EFS contraction amplitudes in each experiment were normalized to a control contraction evoked by 60 mM K^+^ Krebs solution, prior to calculations of means, SEM, or statistical analysis. The addition of TTX (1 μM) eliminated EFS contractions (30 V, 30 Hz), and they were not restored upon the addition of 20 μM E4031 (Figure 5b.ii).

### 3.5 Kv11.1 blockers on contractions of a denuded bladder

**FIGURE 6:**
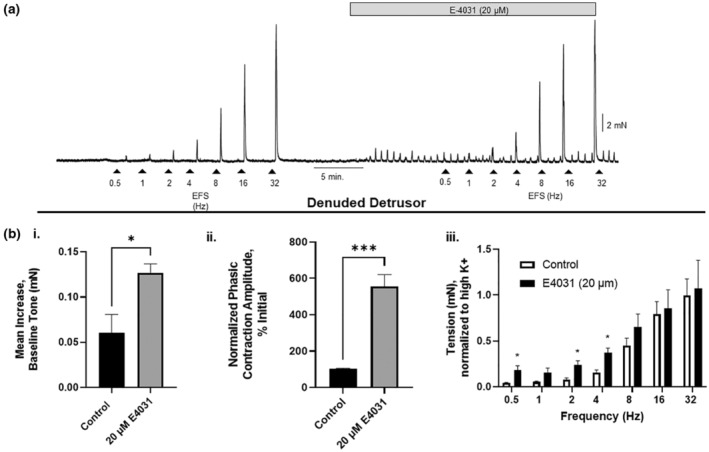
Effect of E‐4031 on contractile activity of a denuded bladder. (a) Electric field stimulation (EFS) of a denuded bladder across a range of frequencies (0.5, 1, 2, 4, 8, 16, 32 Hz). The protocol was conducted before and after incubation with E4031 (20 μM). (b.i) Mean data indicating an increase in baseline tone in the absence (control) and presence of E4031. (b.ii) Average data of increase in phasic contractile amplitude after the addition of E4031 or in the presence of the vehicle (control) (*t* test). (B.iii) Summary data of EFS‐induced contractions (normalized to K+ (60 mM). *p < 0.05, ***p < 0.0001, *N* = 5, error bars represent standard error of the mean).

The bladder was denuded in order to test the efficacy of E4031 on phasic and EFS contractions in the absence of the mucosa (Figure 6a). There was still a significant increase in baseline tone (+0.13 ± 0.01, *N* = 5, *p* < 0.05) (Figure 6b.i) and phasic contraction amplitude, relative to control (556.3% ± 63.9, N = 5, p < 0.001) in a denuded bladder (Figure 6b.ii). E4031 (20 μM) induced a significant increase in EFS contraction amplitude across low frequencies (0.5, 2, 4 Hz; *p* < 0.05, *N* = 5), but had no effect at higher frequencies (Figure 6b.iii). EFS contraction amplitudes were normalized to a control contraction induced by 60 mM K^+^ Krebs solution before calculating means and SEM.

### 3.6 The effect of Kv11.1 activators on EFS‐evoked and spontaneous contractions

**FIGURE 7:**
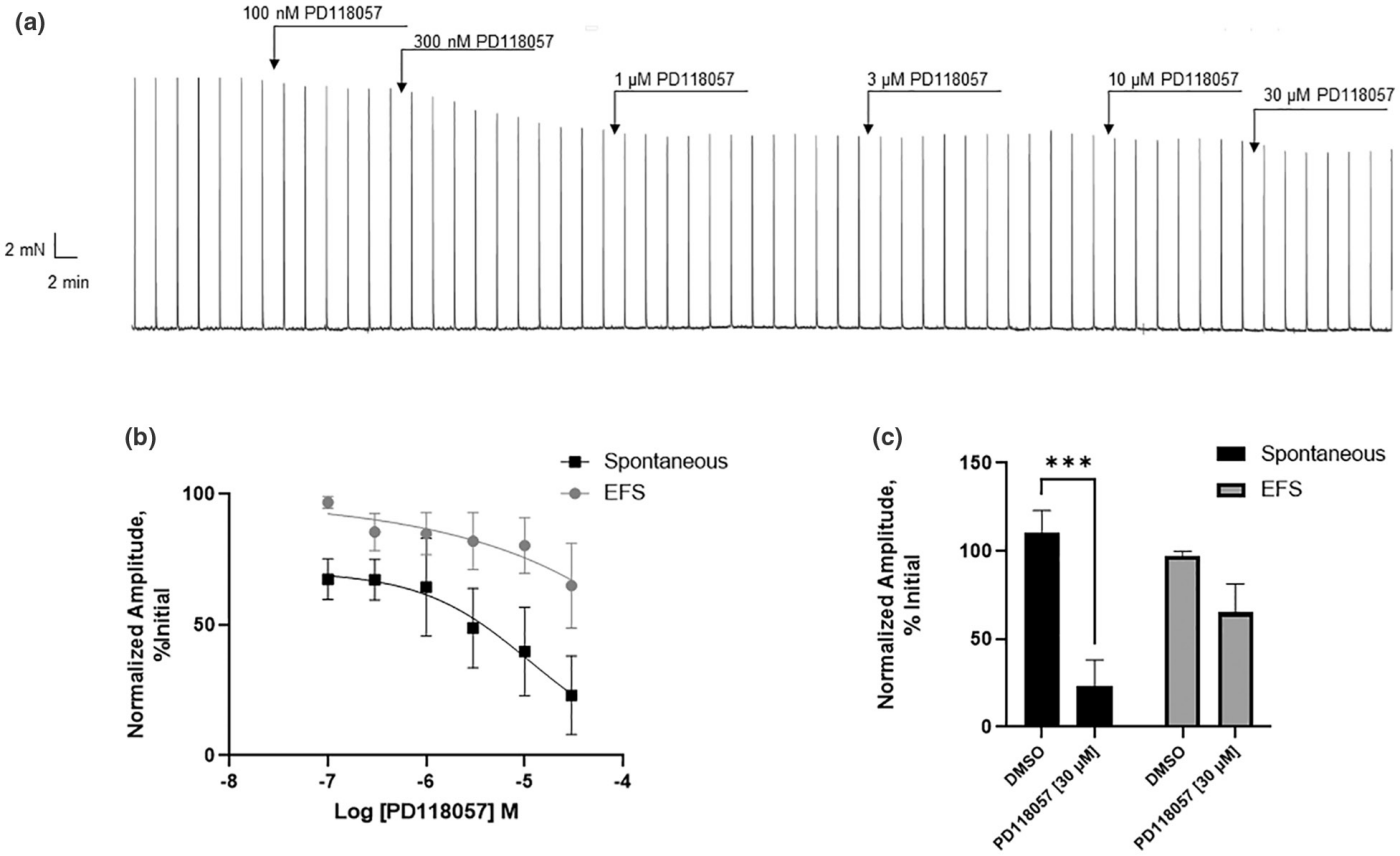
Effect of the Kv11.1 activator, PD118057 on the amplitude of spontaneous and EFS (electric field‐stimulated) contractions (20 Hz). (a) Representative trace of cumulative concentration of PD118057. (b) Normalized amplitudes of spontaneous and EFS contractions at increasing concentrations of PD118057. (c) Normalized amplitudes of spontaneous and EFS contractions in response to PD118057, relative to the vehicle. *N* = 5, Error bars represent standard error of the mean, ***p < 0.001. Statistical analysis using two‐way analysis of variance.

The effects of two structurally dissimilar Kv11.1 activators PD118057 and NS1643 (Casis et al., 2006; Zhou et al., 2005) were assessed on spontaneous and nerve‐evoked contractions. PD118057 significantly reduced spontaneous contractions to ~40% of basal levels (*N* = 5, *p* < 0.05) (Figure 7). NS1643 also reduced spontaneous contractions but this did not reach significance (not shown). Both NS1643 and PD118057 had no significant effect on nerve‐evoked contractions.

### 3.7 Comparison of BK and Kv11.1 blockers on spontaneous activity

**FIGURE 8:**
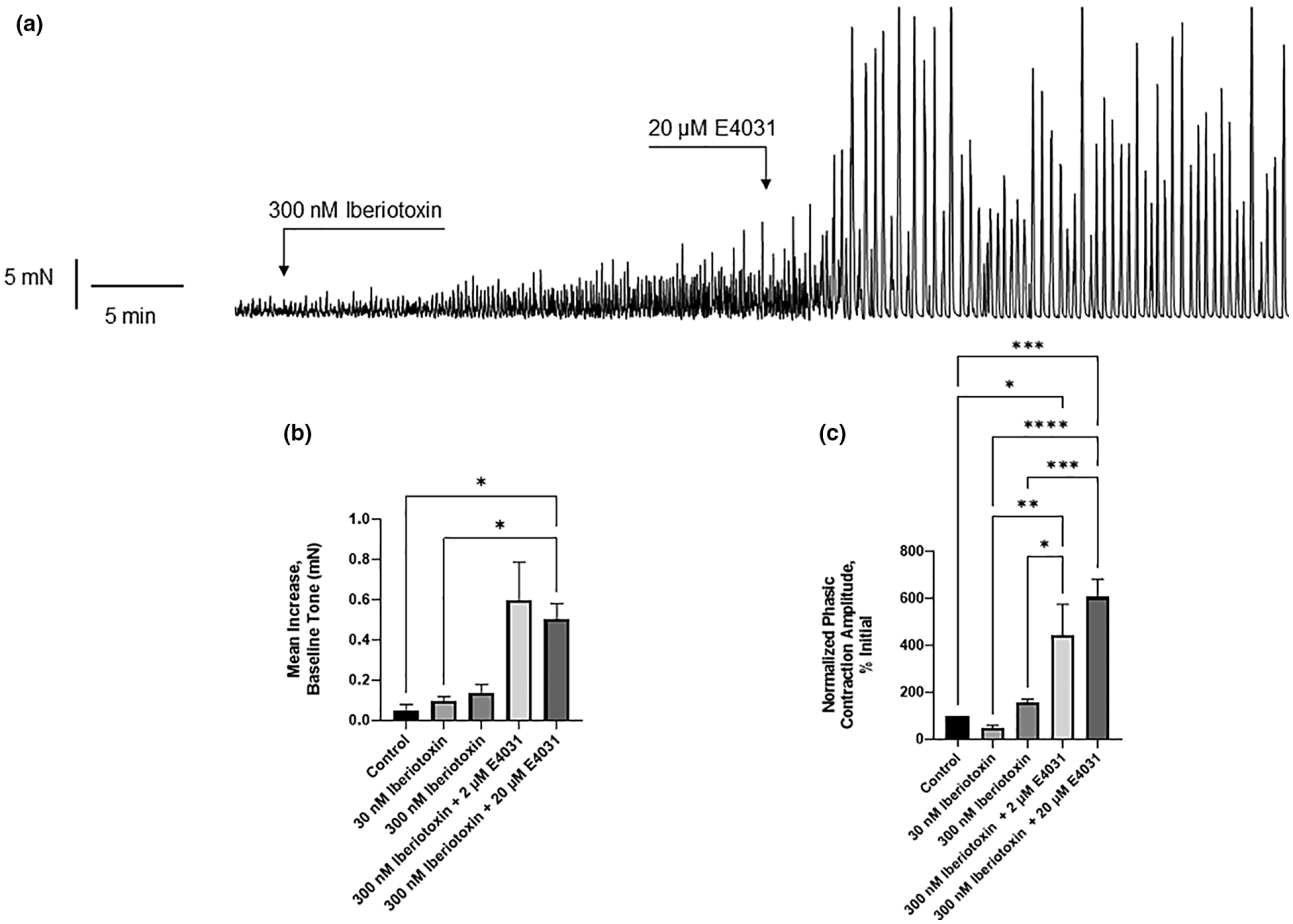
Characterization of baseline tone and phasic contraction amplitude in the presence of BK channel inhibitors in the presence and absence of E4031. (a) Representative trace of basal tone and phasic contraction amplitudes after the addition of 20 μm of the Kv11.1 inhibitor, following preincubation with 300 nM of the BK inhibitor iberiotoxin. (b) Maximum increase in baseline tone in the presence of iberiotoxin with and without E4031 (*N* = 5). (c) Percent increase in the amplitude of phasic contractions compared with control, in the presence of the Kv11.1 inhibitor, E4031 with and without prior incubation with iberiotoxin (*N* = 5). *p < 0.05, **p < 0.01, ***p < 0.001, ****p < 0.0001, error bars represent SEM. Statistical testing using one‐way ANOVA (N = 5, one‐way ANOVA, **p < 0.01, error bars represent SEM). ANOVA, analysis of variance; SEM, standard error of the mean.

BK channels are considered to have significant roles in bladder SMC physiology (Meredith et al., 2004; Petkov, 2014) and have been studied extensively in tissue myography and single‐cell patch‐clamp recordings (Cotton et al., 1996; Herrera & Nelson, 2002). Here, we investigated the impact of blocking BK and Kv11.1 channels on bladder spontaneous contractions (Figure 8). Application of the BK channel blocker, iberiotoxin (IbTX, 300 nM) increased the amplitude of spontaneous contractions amplitude by 156.6 ± 15.90% (SEM, *N* = 5) that was associated with a small (0.13 mN ± 0.04) increase in basal tone (Figure 8b,c). Subsequent addition of E4031 (20 μM) augmented the amplitude of spontaneous contractions (606.3 ± 76.42%, N = 5), similar to the increase induced by E4031 (20 μM) alone (~634% ± 82.89). Application of E4031 to tissues incubated in IbTX increased resting tension (0.50 ± 0.0.078 mN) to a level similar to that seen with E4031 (20 μM) alone (~0.5 ± 0.09) (Figure 8b,c).

### 3.8 Isoprenaline‐mediated relaxations are affected by Kv11.1 blockade

**FIGURE 9:**
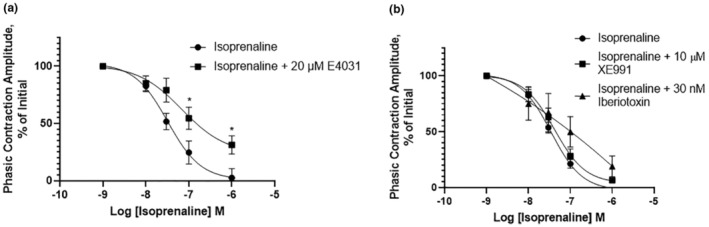
The effect of isoprenaline in the presence of potassium channel inhibitors. (a) Decrease in phasic contraction amplitude of detrusor muscle strips upon treatment with Isoprenaline. The relaxation effect of Isoprenaline is significantly counteracted upon pretreatment with E4031 (b) Preincubation with either XE991 or Iberiotoxin did not significantly alter the adrenergic effect of Isoprenaline (*N* = 5), *p < 0.05; error bars represent standard error of the mean, statistical analysis using two‐way analysis of variance, with Bonferroni multiple comparison test.

Stimulation of cAMP‐linked β‐adrenoceptors (predominantly β3) reduces detrusor smooth muscle contractility. As Kv11.1 channel activity is enhanced by cAMP‐dependent protein kinase A (e.g., Chen et al., 2010) we speculated if Kv11 channels contributed functionally to β‐adrenoceptor‐mediated responses. Application of the general β‐adrenoceptor agonist isoprenaline produced a concentration‐dependent inhibition of spontaneous activity that had been augmented by the addition of 20 mM KCl (Figure 9a). The presence of E4031 (2 μM) attenuated the inhibitory effect of isoprenaline markedly (Figure 9a). BK and Kv7 channels have also been implicated in relaxations produced by agonists of cAMP‐linked receptors (e.g., Huang & Kwok, 1997; Stott et al., 2018; Van der Horst et al., 2020). In contrast to the effect of E4031 on isoprenaline‐mediated responses, neither IBTX (100 nM) nor the Kv7 channel blocker XE991 (10 μM) altered the inhibitory effect of isoprenaline (Figure 9b). These data suggest that Kv11.1 channels provide a functional endpoint for β‐adrenoceptor‐related actions on bladder contractility.

## 4 DISCUSSION

This study demonstrated that male rat bladders expressed both known variants of *Kcnh2* but not *Kcnh6* or *Kcnh7*. Immunocytochemistry established that the *Kcnh2* expression product, Kv11.1, was located predominantly at the cell membrane and patch‐clamp experiments demonstrated potassium currents with distinctive hooked kinetics sensitive to the selective Kv11.1 blocker, E4031, were present in bladder SMCs. In tension recordings, the blockade of Kv11.1 by two structurally different agents, E4031 and dofetilide, enhanced the contractile activity of the detrusor. In addition, Kv11.1 blockers augmented the contractile response of the muscarinic receptor agonist carbachol and attenuated the relaxant effect of the β‐adrenoceptor agonist, isoprenaline.

Blockade of Kv11.1 channels with two structurally different agents enhanced the spontaneous contractile activity of rat bladders similar to preliminary data published by Imai et al. (2001) in guinea pig bladder. These effects far exceeded those produced by blockers of BK_Ca_ and Kv7 channels (IBTX and XE991, respectively) and were manifest as an increase in the amplitude and duration of spontaneous events leading to a tonic increase in tension. These data are consistent with Kv11.1 channels contributing to the resting membrane potential and repolarization of the action potential associated with spontaneous contractions (Hashitani & Brading, 2003). These pronounced effects of Kv11.1 blockers have also been observed in opossum esophagus, mouse gall bladder, equine and human jejunum, bovine epididymis, mouse and human myometrium (Akbarali et al., 1999; Farrelly et al., 2003; Greenwood et al., 2009; Lillich et al., 2003; Mewe et al., 2008; Parkington et al., 2014; Parr et al., 2003). In many tissues, the application of Kv11.1 blockers can convert mechanically quiescent smooth muscle to actively contracting tissues suggesting the contractile activity of the tissue may be dictated by the relative dominance of Kv11.1 channels. As this study demonstrated, the effects of Kv11.1 blockers on bladder contractility were mediated via detrusor SMCs, as even in the absence of a mucosa, phasic contraction amplitude was still significantly elevated. Moreover, inhibition of neuronal activation via TTX did not prevent the effect of E4031 on detrusor phasic contractility. However, this does not rule out that E4031 may also be affecting urothelial, interstitial cells, or neuronal depolarization and remains to be further investigated in future studies. Of note, Kv11.1 channels have been identified in neurons and they are involved in mediating the neuronal resting current (e.g., Sanchez‐Conde et al., 2022).

Moreover, Kv11.1 protein was identified in the plasma membrane of α‐smooth muscle actin‐positive cells and E4031‐sensitive currents with the distinctive voltage‐dependent kinetics that is inherent to the channels were recorded from isolated SMCs. Kv11.1 currents have previously been recorded in SMCs isolated from opossum esophagus, mouse portal vein, and myometrium from mice and humans (Akbarali et al., 1999; Greenwood et al., 2009; Ohya, Horowitz, et al., 2002; Parkington et al., 2014; Yeung & Greenwood, 2007). The voltage‐dependent kinetics recorded in the present study were remarkably similar to those recorded in the other cell types. The combined data suggest that Kv11.1 is expressed by the detrusor SMCs and it contributes markedly to the regulation of smooth muscle contractility. There are two known rat isoforms of Kv11.1, previously known as erg1a and 1b. With respect to erg1a, erg1b lacks 373 amino acids and has a different N‐terminus. The N‐terminal region of erg1a forms the protein domain responsible for the characteristic deactivation kinetics of the channel, and the so‐called “PAS domain,” which defines the ether‐a‐go‐go subfamily of voltage‐gated potassium channels. Erg1a and Erg1b co‐assemble in the endoplasmic reticulum to form a tetrameric channel that mediates the “native” current in cardiac myocytes. Heteromeric erg1a/1b channels show faster activation, deactivation, and recovery from inactivation than channels formed by erg1a alone, a characteristic consistent with the lack of the N‐terminal region responsible for the deactivation kinetics in erg1b. On the other hand, erg1b modulates channel trafficking and response to hormones and other endogenous agonists (Vanderberg et al., 2012). While the molecular composition of the Kv11.1 channel in the bladder remains to be defined the relative lack of the shorter isoform suggests a heterotetramer is unlikely.

In addition to verifying the expression of *Kcnh2* and characterizing the role of Kv11.1, we also compared its effect to the large conductance voltage‐activated and Ca^2+^‐activated (BK) channels. The BK channel is activated by both voltage and Ca^2+^ and is well expressed in the detrusor (Petkov, 2011). Consistent with previous studies application of the BK channel blocker, IBTX, enhanced spontaneous activity; however, its impact was substantially smaller than that of the Kv11.1 inhibitors. Application of E4031 in the continued presence of iberiotoxin produced a striking enhancement of contractile activity reinforcing the view that Kv11.1 channels provide considerable capacity for suppressing bladder contractions and are major determinants of resting activity. It should be noted that detrusor SMCs also express a variety of other K+ channels, which collectively modulate and contribute to resting membrane potential and repolarization (Malysz & Petkov, 2020). For example, SK channels, which are Ca^2+^ sensitive but voltage insensitive, are activated after the hyperpolarization phase and are present in much less abundance compared with BK channels. Pharmacological inhibition of these channels is associated with increased phasic contraction frequency (Herrera et al., 2003). Other channels that have been detected on the DSM include inward‐rectifying ATP‐sensitive K+ channels (K_ATP_). These channels are activated at low intracellular concentrations of ATP, whereas they are inhibited at higher concentrations. Future studies will elaborate on the interplay of Kv11.1 channels with other K channels expressed in the bladder.

E4031 also enhanced carbachol‐induced contractility by ~25%, suggesting that Kv11.1 is not only important in regulating baseline tone and spontaneous activity but also limits the amplitude of receptor‐operated contractions. Our finding that EFS‐mediated, neurogenic contractions could also be augmented by E4031, shows that Kv11.1 activity may provide a protective limit to bladder contraction amplitude during micturition. This would maintain metabolic homeostasis and also provide a further tuning mechanism for appropriate contraction amplitude. Such a mechanism might be expected to react to the relaxation of bladder smooth muscle and this was tested in the present study using, isoprenaline, a nonspecific β‐adrenergic receptor agonist, that induced a concentration‐dependent relaxation, almost abolishing spontaneous contractions. Notably, E4031 but not blockers of BK or Kv7 channels blunted the relaxation effect of isoprenaline. Activation of β‐adrenergic receptors stimulates Kv11.1 channels via a cAMP‐PKA mechanism (Kiehl, 2000; Vandenberg et al., 2012); furthermore, Kv11.1 protein contains numerous protein kinase A (PKA) phosphorylation sites within its intracellular domain, and, within cardiac myocytes, the interaction activates the channel enabling greater channel activation (Kiehl, 2000; Vandenberg et al., 2012). Thus, Kv11.1 may also have an important role in mediating β‐adrenergic receptor bladder relaxation.

Dual actions of Kv11.1 blockers enhancing contraction (spontaneous, neurogenic, and muscarinic receptor‐operated) and impairing relaxation (β‐adrenergic receptor‐operated) reveal the versatility of Kv11.1‐mediated signaling in bladder physiology. While the complexity of mechanisms is not yet understood, Kv11.1 function is a promising area of study. Bladder dysfunction and lower urinary tract symptoms are common and represent many diverse pathophysiologies. Many targets have been identified and investigated for clinical translation, notably, drugs‐targeting muscarinic receptors and β3‐adrenergic receptor agonists are used clinically. BK channel activators were considered to be promising to reduce detrusor overactivity; however, the finding that BK is underexpressed in the neurogenic bladder impeded further development. Interestingly, gene therapies to restore BK channel expression in the neurogenic bladder have been in clinical trials and may bring benefits to patients. With the known key function of Kv11.1 channels in cardiac myocytes (Sanguinetti et al., 1996), their importance is especially notable in individuals with congenital defects in Kv11.1 channels, where resulting cardiac arrhythmia may lead to sudden death. Mutations in *KCNH2* are responsible for approximately 25% of hereditary arrhythmias that present with QT interval prolongation (Sanguinetti et al., 1996); it is not known whether this has consequences for bladder function. All drugs are screened for activity against Kv11.1 to avoid adverse cardiac effects and this might suggest that Kv11.1 could not be progressed as a target to treat bladder dysfunction. Research directed at the characterization of bladder Kv11.1 protein structure, subtypes and pharmacological profile compared with cardiac channels may provide the opportunity to progress bladder‐selective drugs. It is also important to discover whether existing bladder dysfunction treatments have actions on bladder Kv11.1 in addition to their known targets.

In conclusion, the findings of the study reveal functional expression of *kcnh*2 transcript as Kv11.1 ion channels in rat bladder smooth muscle. Pharmacological modulators reveal that Kv11.1 are key determinants of bladder contractility and may represent a more powerful brake on excitability than BK or other Kv channels. The findings show the participation of Kv11.1 in spontaneous, neurogenic, and receptor‐mediated contractions, suggesting a central role in bladder physiology. It is not yet known whether *kcnh*2 mutations are linked with bladder dysfunction or whether Kv11.1 modulators could restore normal contractility if developed with a cardiac‐safe profile; we highlight this as an important area for further research.

## AUTHOR CONTRIBUTIONS

Vincenzo Barrese performed molecular research. Alice Linden, Karen D. McCloskey, Sarah McDowell, and Zena Wehbe performed functional research. Zena Wehbe performed immunocytochemistry. Oleksander Povstyan conducted electrophysiology research. Zena Wehbe and Iain A. Greenwood wrote the manuscript. Iain A. Greenwood designed the research study. All authors contributed to the manuscript and approved the submitted version.

## FUNDING INFORMATION

There are no funders for this research.

## CONFLICT OF INTEREST STATEMENT

The authors declare that they have no conflict of interest.

## DECLARATION OF TRANSPARENCY AND SCIENTIFIC RIGOR

We declare that our paper adheres to the principles for transparent reporting and scientific rigor of preclinic research as stated in the BJP guidelines for Natural Products Research, Design and Analysis, Immunoblotting and Immunochemistry, and Animal Experimentations, as recommended by funding agencies, publishers, and other organizations engaged with supporting research.

## ETHICS APPROVAL STATEMENT

Animals used in the following investigation were handled in strict accordance with the Animal (Scientific Procedures) Act 1986.
